# Perspectives on the Therapeutic Effects of Pelvic Floor Electrical Stimulation: A Systematic Review

**DOI:** 10.3390/ijerph192114035

**Published:** 2022-10-28

**Authors:** Ana Lúcia Carneiro Sarmento, Bruno Silva Sá, Andreanne Gomes Vasconcelos, Daniel Dias Rufino Arcanjo, Alessandra Durazzo, Massimo Lucarini, José Roberto de Souza de Almeida Leite, Hugo Alves Sousa, Selma Aparecida Souza Kückelhaus

**Affiliations:** 1 Research Center in Applied Morphology and Immunology (NuPMIA), Faculty of Medicine (FM), University of Brasília (UnB), Brasília 70910-900, DF, Brazil; 2 Department of Biomedicine, University Centre of the Federal District (UDF), Brasília 70390-045, DF, Brazil; 3 Laboratory of Functional and Molecular Studies in Physiopharmacology (LAFMOL), Department of Biophysics and Physiology, Federal University of Piauí (UFPI), Teresina 64049-550, PI, Brazil; 4 CREA-Research Centre for Food and Nutrition, Via Ardeatina 546, 00178 Rome, Italy

**Keywords:** pelvic floor electrical stimulation, muscle contraction, pelvic floor contraction

## Abstract

Pelvic, perineal, and nervous lesions, which derive principally from pregnancy and childbirth, may lead to pelvic floor dysfunctions, such as organ prolapses and lesions in the nerves and muscles due to muscle expansion and physiology. It is estimated that 70% of women affected by this clinical picture have symptoms that do not respond to the classical treatments with antimuscarinic and anticholinergic drugs. Therefore, resorting to efficient alternatives and less invasive methods is necessary to assist this public health problem that predominantly affects the female population, which is more susceptible to the risk factors. This study aimed to perform an updated and comprehensive literature review focused on the effects of pelvic floor electrical stimulation, considering new perspectives such as a correlation between electric current and site of intervention and other molecular aspects, different from the present reviews that predominantly evaluate urodynamic aspects. For that purpose, PubMed and ScienceDirect databases were used to perform the search, and the *Methodi ordinatio* method was applied. With well-researched therapeutic effects, electrical stimulation induced promising results in histological, nervous, and molecular evaluations and spinal processes, which showed beneficial results and revealed new perspectives on ways to evoke responses in the lower urinary tract in a non-invasive way. Thus, it is possible to conclude that this type of intervention may be a non-invasive alternative to treat pelvic and perineal dysfunctions.

## 1. Introduction

During pregnancy and, principally, at childbirth, pelvic floor nerves and muscles reach alterations beyond the normal physiology, which are determinant for favoring childbirth but also induce pelvic and perineal muscle dysfunctions [1]. In this aspect, muscle and nerve expansion may induce the prolapse of pelvic organs, perineal lesion, and stress urinary incontinence, besides damaging the pudendal nerve, which may reflect on the innervation of the pelvic floor [2].

Stress urinary incontinence reaches 54.3% of pregnant women and is characterized by involuntary urine loss caused by abdominal pressure without detrusor muscle contraction [3]. In addition, muscular and vascular components contribute to urethral wall elasticity and pelvic floor innervation, but they also contribute to the conditions associated with this clinical picture [4]. Lower urinary tract dysfunction caused by neurological disorders, such as medullary lesions, sclerosis, and cerebrovascular accident, represents another clinical condition affecting urination efficiency, detrusor, and urethral sphincter contraction, bladder storage volume [5].

In addition, it is estimated that spontaneous vaginal births are responsible for first- and second-degree perineal lacerations in 38% of primiparous women and 36% of multiparous women. They have also been associated with maternal morbidity, including pain, flatulence, fecal incontinence, and dyspareunia [6]. Episiotomy represents also an impacting factor during postpartum once this obstetric intervention is associated with higher rates of perineal pain and lesion, which, according to the degree of perineal exposure, may lead to sphincter lesions, inducing rectal complacency loss, collaborating for fecal incontinence [7].

In men, the pelvic floor muscles such as bulbocavernosus and ischeocavernosus that play an important role in urinary continence and erection, can lose contraction force in individuals after radical prostatectomy due to prostate cancer [8]. Cases of urinary incontinence after radical prostatectomy can last up to 5 years in 65% of the male audience, being attributed to the deficiency of the sphincter, in the contraction force of the pelvic musculature and bladder compliance, which impacts on functional aspects that negatively affect the quality of life after surgical procedure [9]. However, the electrical stimulation of pelvic skeletal muscles proves to be a predictive factor for the recovery of contraction force, detrusor hyperactivity and erectile function in patients, in addition to increasing urethral closure [10].

The therapeutic approaches applied for treating stress urinary and fecal incontinence depend on/is affected by the patient clinical severity. Therapeutic interventions are preferentially carried out by applying non-invasive methods in cases without structural or functional lesions, while invasive approaches are utilized in case of severe muscular lesions or dysfunctions, where non-invasive treatment was not responsive [11]. Among the most conservative interventions, dietary re-education and the administration of drugs with anti-motility and constrictor effects, are usually the first choices to treat incontinence [12]. In the most serious cases, which usually do not respond to the primary interventions, the methods applied include injections with fillers in the intersphincteric and submucous spaces of the anal canal, radiofrequency (aiming to cause fibrosis in the anal canal), sphincteroplasty, perianal reconstruction or artificial sphincter. However, some these procedures have temporary therapeutical effects [13].

The recommendation of electrical stimulation as a treatment for musculoskeletal disorders of the pelvis and perineum are diverse. It has been used in clinical practice for pain mitigation, muscle rehabilitation, treatment of motor/conscious disorders, wound healing, and drug administration [14]. In addition, it has also been prescribed for the conservative treatment of urinary incontinence (UI) by evoked pelvic floor muscle training [15]; the studies by Jha et al. (2018) [16] showed its benefits in the treatment of UI associated with sexual dysfunction. Although electro stimulation is used as a therapeutic agent for disorders of the pelvis and perineum, there is a lack of consistency in the protocols. One reason for this is that basic information about its effect on pelvic and perineal musculature is based on clinical findings and theoretical assumptions and not based on histological findings.

Regarding histological effects, literature reports the benefits of electrical stimulation applied to the muscles of the anorectal region. This technique promotes hypertrophy and hyperplasia of muscle fibers, greater production of collagen fibers and inhibit apoptosis [3,4,5,6,7,8,9,10,11,12,13,14,15,16,17]. Additionally, electrical stimulation regenerated ovoid axonal fibers with myelination defects and Wallerian degeneration in vaginal nerves and tissues, increasing the diameter of the axons and myelin thickness [18]. The urodynamic effects of electrical stimulation include the multifactorial recovery of detrusor involuntary contraction, maximal bladder pressure, besides reducing urine loss and nocturia, with minimal side effects and are therefore the first choice before administration of anticholinergic and antimuscarinic drugs [19].

In this aspect, electrical stimulation is a potential non-invasive alternative for treating pelvic floor and perineal dysfunctions, transmitting electrical current in different intensities to stimulate muscles and nerves, to improve their contraction and flexibility, besides activating inert nerves [18]. Thus, this systematic review aimed to evaluate the scientific literature about electrical stimulation for controlling pelvic floor and perineal dysfunctions through the therapeutic effect induced by the stimulation of nerves, muscles, and spinal processes.

## 2. Materials and Methods

This study was performed according to the pre-specified PRISMA statement, which was applied to assist the selection of studies, data extraction, and results analysis. It is a systematic review of literature, with quantitative and descriptive characteristics, carried out by analyzing studies published in English and available in databases, i.e., PubMed (Medline) and ScienceDirect. The search was performed with the following descriptors: “electrical stimulation” AND “pelvic muscle” AND “muscle contraction”, covering the period of 2017–2022 to cover new evaluations on electrostimulation and new discoveries that enable new perspectives on the mechanisms and therapeutic effects of the intervention.

The pre-selection carried out starring from the title analysis and abstract reading, included original experimental research, published during the last five years, written in English, and fitting the proposed theme (effects of the electrical stimulation on the pelvic floor). In Table 1, inclusion and exclusion criteria of the study are reported. Furthermore, the reference manager Zotero (version 6-https://www.zotero.org/download/, accessed on 25 July 2022) was used to manage the bibliographic data and help to exclude duplicated articles as instructed in Pagani et al., 2018 [20].

After the pre-selection, the eligible articles were submitted to the Methodi Ordinatio equation proposed by Pagani et al. (2018) [20], aiming to select the final bibliography to be reviewed. After applying this method, which considers the impact factor (IF), the number of citations (Ci), and publishing year, the articles were sorted in descending order of InOrdinatio value, described in Equation (1), selecting the articles with higher scientific quality, according to the previously mentioned parameters. More information about the application and scientific quality of the equation available in Pagani et al. (2018) [20].

(1) $$\mathrm{InOrdinatio}=\left(\frac{\mathrm{IF}}{1000}\right)+\left(\alpha \;\left(10-\left(\mathrm{Research}\;\mathrm{year}-\mathrm{Publish}\;\mathrm{year}\right)\right)\right)+\left(\mathrm{Ci}\right)$$

Equation (1). InOrdinatio—IF: impact factor; α: constant varying from 0 to 10, value attributed by the researcher; Ci: number of citations.

To perform the calculations of the InOrdinatio equation, we obtained the IF values with the aid of Journal Citation Reports (JCR), the Ci with Google Scholar, and the publishing year with Zotero reference manager. After calculating the InOrdinatio value, the articles were organized in a table using Microsoft Excel^®^, excluding the articles with no InOrdinatio values established by the software. After selecting the final bibliography, the articles were fully read to gather information, such as electrical stimulation site, electrical current charge, and the effects of post-electrical stimulation in humans and animals.

## 3. Results

**Figure 1:**
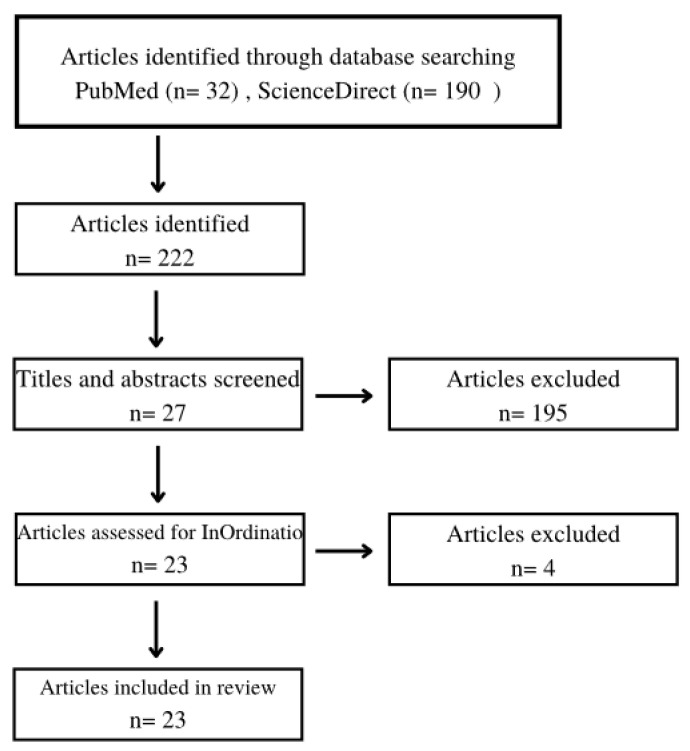
Flowchart of the study selection process.

A total of 222 articles were found in the literature search. After applying the eligibility criteria, the pre-selection process (title and abstract reading) provided 27 articles. These articles were submitted to the Methodi Ordinatio equation, considering the scientific quality of each work, and the final selection resulted in 23 studies, which composed the bibliographic collection of this review. The flowchart of the study selection process was reported in Figure 1. Based on the pre-selection criteria, we excluded 195 articles that did not suit the theme. Another four articles were excluded during the final selection process considering the InOrdinatio value.

After the study selection process, 23 articles were included in our study and classified according methodological InOrdinatio classification as represented in Table 2. The classification followed the InOrdinatio value, which equates the impact factor (IF), Citation Number (Ci), and publishing year. According to Pagani et al. (2018) [20], this method classifies the quality of the studies by analyzing relevant issues. The citation number, for example, indicates the study appreciation by the scientific community. The publishing year, in turn, suggests how current the methods applied in the study and the published data are. Finally, the impact factor demonstrates how important that study is for the society. Thus, this method was applied to certify the selection of a high-quality bibliographic collection by the methodological measurement and classifying the scientific journals where the studies were published.

**Figure 2:**
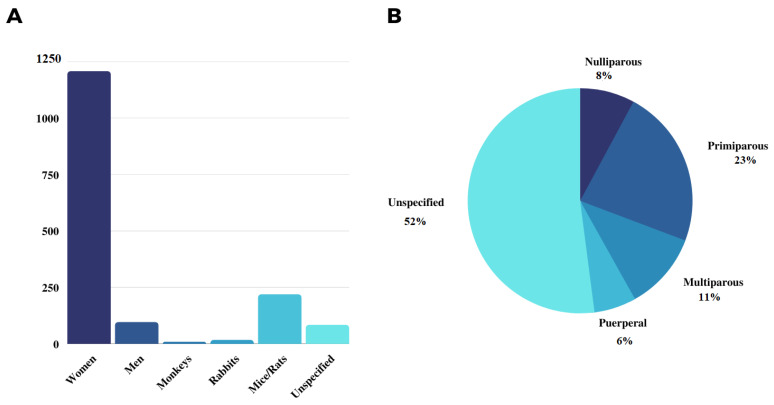
Studied populations (**A**) and female classification (**B**) found in the analyzed studies.

Regarding the studied populations, the results demonstrated heterogeneity between human and animal populations. Articles [16,21,22,23,24,25,26,27,28,29,30,31,32,33,34] comprised studies that investigated the therapeutic effects of electrical stimulation on pelvic floor dysfunctions in humans, totaling 1303 participants. From these, only the research performed by [25] included men in the study population, which investigated 96 patients with urinary incontinence post-radical prostatectomy. In contrast to the number of humans studied, in vivo experiments using animal models were predominantly characterized by studies in monkeys [5], rabbits [18], mice, and rats [3,17,35], the latter two representing the highest samples, with 219 individuals (Figure 2A). Female models, in turn, were predominantly composed of primiparous women, with 328 individuals studied, in contrast to 113, 160 and 87 nulliparous, multiparous, and puerperal women, respectively (Figure 2B), and the studies performed did not specify the studied population, totaling 747 unspecified individuals.

Considering the electrical intensity and the intervention site, we found that 16 studies (69.6% of the total reviewed bibliography) treated the studied population with electric intensities varying from 20–50 Hz. Additionally, no differences were observed regarding the electrical intensities applied to humans compared to animals, except for the study by [5], which used the intensity of 1 Hz in *Rhesus* monkeys, aiming to evoke an answer in the lower urinary tract. Regarding the most approached regions to perform electrical stimulation, 14 studies reported a direct approach to the anogenital region (vagina, penis and anus), while other 9 studies carried out the induction in nerves, i.e., pudendal, tibial, bulbospongiosus, and pubococcygeus nerves. The relation between the site and intensity of the application performed by each study are reported in Table 3.

By analyzing the effects, therapeutic properties of electrical stimulation, associated or not with pelvic and perineal exercises, were investigated in 17 studies with humans and 6 studies with animal models. The reviewed studies demonstrated different results between human and animal models, but it is also possible to observe that these results corroborate each other because the quantitative results in vivo and in vitro evaluations in the animal population, such as an increase in the number of collagen and hypertrophy, corroborate the clinical results of the human population, as a greater strength of contraction of the muscle. Human studies evaluated clinical conditions, i.e., pain, pelvic organ prolapses, and incontinence by urodynamic criteria. In animal studies the results were limited to the morphological characteristics of tissues and nerves submitted to the treatment, besides the investigation of signaling pathways by analyzing the expression of molecules possibly involved in the observed effects Table 4 describes. Therapeutic effects of electrical stimulation associated or not with pelvic and perineal exercises reported in documents from literature search.

## 4. Discussion

Concerning the studied population distribution (Figure 2A), recruitment of the female population predominates over the male population may indicate the preference of that group in the search for therapeutic alternatives in public health. It may also be related to the risk of developing pelvic floor dysfunctions, which is higher in women between 30 and 60 years old, as well as puerperal women [29,37], despite the high incidence of urinary incontinence in men submitted to radical prostatectomy (69–98%) [25]. According to Ignácio et al., 2022 [24], 70% of women with pelvic floor dysfunction derived from pregnancy, childbirth, or physical effort with an increase in abdominal pressure present inadequate contraction of pelvic floor muscles, and 97% have weak contractions. These are etiologic factors for pelvic organ prolapses and anal incontinence. Thus, a higher number of women recruited in the studies may be justified by the necessity to find less invasive alternatives effective for treating this potentially susceptible group.

By evaluating the female classification (Figure 2B), 30.8% comprised primiparous and nulliparous women were found, while 11.1% of this classification comprised multiparous. Despite these findings, Hernandez-Reynoso et al., 2021 [18] reported that multiparous and elderly women constitute the ideal model for studying the effects of electrical stimulation on the pelvic floor. In this model, pelvic dysfunctions, especially urinary and fecal incontinence, derive from parity and aging. Compared with nulliparous women, these conditions are characterized by muscle and nerve strain, muscle contraction without synchronization, low urethral pressure, and bladder efficacy, besides reduction in the action potential required for muscle contraction. Regarding animal models, although the number of studies with mice/rats is higher than those with rabbits and monkeys (Figure 2A), Hernandez-Reynoso et al., 2021 [18] emphasize that rabbits may be most suitable than mice for studying electrical stimulation aiming translational studies related to human pelvic muscles, because rabbits have well-developed pelvic floor muscles, in contrast to mice, which have vestigial muscles. In this aspect, characteristics, such as the study population and model, can provide populational samples with relevant impacts to the final application of the proposed intervention.

In the study performed by Min et al., 2017 [37] the vaginal dilation (VD) group had a significant decrease in the expression of calcium channel subtypes, such as type-T Cav3.1 and Cav3.2, compared to the group without intervention (control). After the electrical stimulation, the groups stimulated with 20 and 50 Hz showed the opposite effect, with increased expression of Cav3.1 and Cav3.2 compared to the control group. The highest increase was observed for Cav3.2 after stimulation with 50 Hz [37]. Comparatively, Li et al., 2019 [3], by aiming to identify signaling molecules involved with collagen modulation in electrical stimulation, demonstrated a significant increase in intracellular Ca^2+^ concentration in the L929 cell line. However, this effect was not observed when the cells were exposed to a Ca^2+^-free medium, showing that electrical stimulation does not induce Ca^2+^ expression but may influence calcium ion flux in the cells [3]. Altogether, type-T calcium channels are more likely to be activated with lower membrane potential, favoring the intervention due to low intensity, indicating that the high expression of Cav3.2 reported by Min et al., 2017 [37] may be responsible for the high flux of Ca^2+^ ions observed by Li Y et al., 2019 [3].

Besides, the histological analysis performed by Min et al., 2017 [37] demonstrated that the anterior vaginal wall of the control group had a thick layer of smooth muscle and organized dense connective tissue. In contrast, the group submitted to vagina dilation (simulating vaginal birth conditions) had reduced, fragmented, and disorganized collagen fibers. After the electrical stimulation, treated animals presented an increase in collagen fibers, which was higher in the group stimulated with 50 Hz compared to the group stimulated with 20 Hz. Subsequently, the authors evaluated collagen expression, and the results demonstrated a significant decrease in the expression of collagen I and III proteins and mRNA in the vaginal dilation group. On the other hand, only the VD+ES50 group had a significant increase in the collagen protein expression, which suggests that the threshold of electric current intensity may be responsible for displaying this result in collagen expression and may justify the histological results previously mentioned for the electrical stimulation with 50 Hz, which also demonstrated increased collagen fibers. In addition, de Sousa et al., 2017 [17] demonstrated a 372.1% increase in the median of the mucosa layer thickness, besides a 68.9% and 137.1% increase in the submucosa and muscle thickness, respectively. The same study also demonstrated effects on anus-associated muscles, with a 34.8% increase in muscle fibers, besides a 58% and 30.5% increase in the thickness of internal and external anal sphincter fibers, respectively. These effects corroborate with the reepithelization, hyperplasia, and hypertrophy, besides the biosynthesis of collagen types present in the connective tissue, supporting pelvic muscle and enabling the therapeutic effect after restoration.

The previously mentioned results showed the relevance of collagen levels [16], which impact pelvic floor strengthening, besides Ca^2+^ ion concentration, which is widely distributed and regulate cell proliferation, differentiation, as well as other cell biological activities. These factors seem to be involved with the pathogenesis of pelvic floor dysfunction and in the response to electrical stimulation. Under another perspective, de Sousa et al., 2017 [17] also observed the immunological effect of electrical stimulation on the inflammatory infiltrate in the rectum- and anus-associated connective tissue. Although the qualitative morphological analysis did not show alterations, the quantitative analysis showed an increase in eosinophils, lymphocytes, and macrophages in the electrically stimulated group, corroborating the idea that this method induces cell proliferation and migration towards the lesion by electrotaxis. Following other perspectives, Havton et al., 2019 [5] demonstrated that some variables of the electrical stimulation, such as current and pulse intensity, as well as the site of intervention, could be predictive factors of the therapeutic effect induction of electrical stimulation. Table 3 reported the relation between the site and intensity of the application performed by each study from literature search.

Havton et al., 2019 [5] concluded that the electrical current intensity threshold is responsible for inducing the therapeutic effect of electrical stimulation, as well as the current pulse alteration (mA) and the stimulated site. In the study, nine female Rhesus monkeys were submitted to non-invasive electrical stimulation on the spinal cord, aiming to observe the effects on the low urinary tract. The authors [5] performed a functional map of the spinal cord for each animal, identifying specific sites of the spinal process and demonstrating alterations in the urodynamic aspects. The authors [5] observed that the alternate of pulse intensity and width, modulating the urination reflexes of the animals, provided better urination efficiency (improvement of 32.8 ± 10.4%) and decreased post-void residue (67.6 ± 9.8%) compared to the void efficiency of 18.2 ± 8.8% and post-void residue of 81.4 ± 7.5% observed before the intervention. These results demonstrated that the procedure was a viable and successful method to activate micturition reflexes, supporting a new approach with the functional map of the spinal cord to selectively activate neural networks and promote efficient therapeutic effects on pelvic floor dysfunction [5]. In contrast to this perception, Li T et al., 2018 [34] performed the electrical stimulation at 2.5 and 35 Hz in the studied groups and reported better results with lower electrical current. The authors [35] observed a 50% improvement in the clinical symptoms, besides a higher potential to decrease post-void residual volume and inhibiting detrusor hyperactivity compared to the group that received the electrical stimulation with higher intensity. However, the contrast between the results of these studies does not eliminate the possibility of a correlation between intensity, width, and intervention site in the therapeutic effect of electrical stimulation. Although some studies demonstrated significant effects using intensities higher than 30 Hz, more studies are still necessary to evaluate this perspective in the results observed for electrical stimulation.

The studies [21,27,31] demonstrated the use of electrical stimulation combined or compared with pelvic rehabilitation exercises. The studies [21,27,31] demonstrated significant therapeutic effects concerning pelvic muscle and perineal region strengthening, as well as a satisfactory impact on life quality. The data from studies [21,27,31] was evaluated by the pelvic organ prolapse degree (POP-Q), the degree of incontinence punctuation, pelvic floor muscle strength (Oxford grading), increase in the pressure and duration of vaginal contraction, besides the electrophysiological condition that validated the cicatricial results on pubic symphysis and types II and III muscle fibers strength. These findings corroborate the results obtained by Hwang et al., 2019 [36], who reported the recruitment of types I and III collagen fibers after electrical stimulation. Collagen fibers are relevant to increasing the strength, power, and resistance of pelvic floor muscles and, consequently, influence endopelvic fascia traction. In addition, training pelvic floor muscles supports the bladder neck, reducing urine loss, and interfering with the urethral-detrusor reflexes, which inhibits involuntary detrusor contractions. In contrast to these results, the combination of electrical stimulation and pelvic floor muscle exercises reported in the study of Jha et al., 2018 [16] did not show significant improvement in the pelvic floor of women with urinary incontinence. Once the intensity and the site of the electrical stimulation were not specified in the study, it was not possible to correlate the studies. Although three out of four articles analyzed in the present study reported beneficial effects of the combination between electrical stimulation and pelvic floor exercises, other studies are still necessary to determine if the combination of the techniques provides better results compared to the isolated approaches.

## 5. Conclusions

Non-invasive electrical stimulation has shown promise in the clinical improvement of disorders associated with pelvic floor fragility. The vast majority of studies addressed in this review showed that electrostimulation improves urination control and sexual quality, in addition to providing greater collagen production and maintaining the effectiveness of sphincter contraction.

Despite the clinical findings favorable to electrostimulation, the lack of standardization related to the route of administration of electrostimulation (surface electrode or intravaginal/anal probe) and the power of the electric current may be confounding factors in the analysis of the effectiveness of the technique. Considering the impossibility of performing tissue and cross-sectional studies in humans, it is important to evaluate studies with animal models that have substantially contributed to show that electrostimulation structurally improves connective and muscular tissues. In view of these findings, it is possible to infer that the functional rehabilitation resulting from clinical studies reflects the structural gain caused by electrical stimulation.

Despite lacking standardization, electrical stimulation has great potential in the treatment of pelvic and perineal floor disorders, especially urinary and fecal incontinence, with a positive impact on the quality of life of patients affected by these conditions.

## Figures and Tables

**Table 1:** Inclusion and exclusion criteria of the study.

Criteria	Inclusion	Exclusion
Population	Humans and animals submitted to electrical stimulation against pelvic and perineal dysfunctions.	Pregnant population
Intervention	Electrical stimulation isolated or in combination with pelvic and perineal exercises	Other interventions
Type of study	Randomized and experimental studies	Literature reviews, encyclopedia, case reports, book chapters.
Language	English	Other languages
Year	2017–2022	Other period

Source: Elaborated by the Authors.

**Table 2:** Methodological InOrdinatio classification of the articles found during the search.

Classification	Authors	InOrdinatio Value
[1]	Yang S et al., 2017	68.04
[2]	Jha S et al., 2018	63.36
[3]	Sonmez R et al., 2021	59.92
[4]	Jacomo RH et al., 2020	57.36
[5]	Antônio, F.I et al., 2022	57
[6]	Feng X et al., 2022	52.64
[7]	Del Río-Gonzalez S et al., 2017	51.64
[8]	Havton LA et al., 2019	50.33
[9]	Zhong F et al., 2021	50.1
[10]	Hernandez AG et al., 2021	49.38
[11]	Mallmann S et al., 2020	48.43
[12]	Hwang UJ et al., 2020	48.43
[13]	Li W et al., 2020	47.89
[14]	Elena S et al., 2020	47.49
[15]	Hwang UJ et al., 2019	47.49
[16]	Oliveira MC et al., 2021	47.43
[17]	Li Yang et al., 2019	47.31
[18]	Min J et al., 2017	46.64
[19]	Mateus–Vasconcelos E et al., 2018	46.38
[20]	Ness TJ et al., 2018	46.05
[21]	Brose SW et al., 2018	45.98
[22]	Li T et al., 2018	37.65
[23]	De Souza HA et al., 2017	33.92

**Table 3:** Relation between electric current and site of intervention reported by the reviewed studies.

No.	Authors	Site of Intervention	Electric Current
[1]	Yang S et al., 2017	Vagina	60–80 Hz
[2]	Jha S et al., 2018	Unspecified	Unspecified
[3]	Sonmez R et al., 2021	Tibial nerve	20 Hz
[4]	Jacomo RH et al., 2020	Tibial nerve and para-sacral region	10 Hz
[5]	Antônio, F.I et al., 2022	Intravaginal	50 Hz
[6]	Feng X et al., 2022	Pudendal nerve and transanal region	2.5 Hz
[7]	Del Río-Gonzalez S et al., 2017	Tibial nerve	20 Hz
[8]	Havton LA et al., 2019	Spinal cord	1 Hz
[9]	Zhong F et al., 2021	Intravaginal	35–80 Hz
[10]	Hernandez AG et al., 2021	Bulbospongious and pubococcygeus nerves	2–20 Hz
[11]	Mallmann S et al., 2020	Tibial nerve and para-sacral region	20 Hz
[12]	Hwang UJ et al., 2020	Perivaginal and sacral	25 Hz
[13]	Li W et al., 2020	Intravaginal	50 Hz
[14]	Elena S et al., 2020	Vagina	2.5 T
[15]	Hwang UJ et al., 2019	Perivaginal and sacral	25 Hz
[16]	Oliveira MC et al., 2021	Tibial nerve	20 Hz
[17]	Li Yang et al., 2019	Vagina	50 Hz
[18]	Min J et al., 2017	Vagina	20–50 Hz
[19]	Mateus–Vasconcelos E et al., 2018	Intravaginal	50 Hz
[20]	Ness TJ et al., 2018	Pudendal nerve	10 Hz
[21]	Brose SW et al., 2018	Anogenital region	20 Hz
[22]	Li T et al., 2018	Pudendal nerve and anogenital region	2.5–3.5 Hz
[23]	De Souza HA et al., 2017	Vagina and rectum	50 Hz

Source: Elaborated by the Authors.

**Table 4:** Therapeutic effects of electrical stimulation associated or not with pelvic and perineal exercises.

Author, Reference Number	Effect
Yang S et al., 2017 [21]	Improvement in incontinence score and Oxford grading for pelvic floor muscle strength. Synergic effect when associated with pelvic rehabilitation exercises.
Jha S et al., 2018 [16]	No significant improvement was observed after electrostimulation and physiotherapy against urinary incontinence and sexual dysfunction.
Sonmez R et al., 2021 [22]	Significant reduction in incontinence score, urination frequency, nocturia, number of absorbents used, and life quality obtained by electrical stimulation.
Jacomo RH et al., 2020 [23]	Electrical stimulation had beneficial effects on the treatment of hyperactive bladder, decreasing incontinence and nocturia scores.
Antônio, F.I et al., 2022 [24]	A 36% increase in the ability to contract pelvic floor muscles and improvement in incontinence score after electrical stimulation.
Feng X et al., 2022 [25]	An improvement in the urinary incontinence score was observed in man with urinary incontinence post-radical prostatectomy.
Del Río-Gonzalez S et al., 2017 [26]	Improvement in clinical and urodynamic parameters with durability up to 24 months after electrical stimulation.
Havton LA et al., 2019 [5]	Improvement in the urinary control in a less invasive way.
Zhong F et al., 2021 [27]	Significant improvement in stage I pelvic organ prolapes, life quality, and sexual quality.
Hernandez AG et al., 2021 [18]	Improvement in axonal composition, diameter, and regeneration of pelvic and perianal nerves.
Mallmann S et al., 2020 [19]	Improvement in life quality, discomfort level, and incontinence score.
Hwang UJ et al., 2020 [28]	Improvement in the power, strength, and resistance of pelvic floor muscles, urinary loss, and incontinence score.
Li W et al., 2020 [29]	Improvement in pelvic muscle contraction and muscle strength.
Elena S et al., 2020 [30]	Improvement in the recovery of pelvic floor muscle strength and sexual dysfunction-associated urinary incontinence.
Hwang UJ et al., 2019 [36]	Improvement in sexual function (desire, excitement, orgasm), besides the power, strength, and resistance of pelvic muscles.
Oliveira MC et al., 2021 [31]	Inconsistent results regarding urinary incontinence improvement.
Li Yang et al., 2019 [3]	Electrical stimulation activated collagen expression, increased the intracellular concentration of calcium, and suppressed apoptosis.
Min J et al., 2017 [37]	Electrical stimulation increased maximum bladder capacity and urodynamic aspects. It also increased collagen and calcium channel, assisting the treatment of urinary incontinence.
Mateus–Vasconcelos et al., 2018 [32]	Decrease in urinary incontinence score.
Ness TJ et al., 2018 [35]	Electrical stimulation induced neuromodulatory effects in pelvic sensorial systems treating bladder painful disorders.
Brose SW et al., 2018 [33]	The treatment decreased detrusor hyperactivity and pelvic pain.
Li T et al., 2018 [34]	Improvement in life quality and residual urine volume.
De Souza HA et al., 2017 [17]	Electrical stimulation improved the morphology of rectum and anus tissues with hyperplasia and hypertrophy, which consequently improved the anal sphincter function.

## Data Availability

Not applicable.

## References

[1] Ghaderi F., Bastani P., Hajebrahimi S., Jafarabadi M.A., Berghmans B. (2019). Pelvic floor rehabilitation in the treatment of women with dyspareunia: A randomized controlled clinical trial. Int. Urogynecol. J..

[2] Deng K., Balog B.M., Lin D.L., Hanzlicek B., Song Q.-X., Zhu H., Damaser M.S. (2019). Daily bilateral pudendal nerve electrical stimulation improves recovery from stress urinary incontinence. Interface Focus.

[3] Li Y., Liu C., Li B., Hong S., Min J., Hu M., Tang J., Wang T., Yang L., Hong L. (2019). Electrical stimulation activates calpain 2 and subsequently upregulates collagens via the integrin β1/TGF-β1 signaling pathway. Cell Signal..

[4] Buckley B.S., Lapitan M.C.M. (2010). Prevalence of Urinary Incontinence in Men, Women, and Children—Current Evidence: Findings of the Fourth International Consultation on Incontinence. Urology.

[5] Havton L.A., Christe K.L., Edgerton V.R., Gad P.N. (2019). Noninvasive spinal neuromodulation to map and augment lower urinary tract function in rhesus macaques. Exp. Neurol..

[6] Albers L.L., Sedler K.D., Bedrick E.J., Teaf D., Peralta P. (2006). Factors Related to Genital Tract Trauma in Normal Spontaneous Vaginal Births. Birth.

[7] Saad L.H.C., Coy C.S.R., Fagundes J.J., Ariyzono M.D.L., Shoji N., Góes J.R.N. (2002). Quantificacao da funcao esfincteriana pela medida da capacidade de sus- tentacao da pressao de contracao voluntaria do canal anal. Arq. Gastroenterol..

[8] Laurienzo C.E., Magnabosco W.J., Jabur F., Faria E.F., Gameiro M.O., Sarri A.J., Kawano P.R., Yamamoto H.A., Reis L.O., Amaro J.L. (2018). Pelvic floor muscle training and electrical stimulation as rehabilitation after radical prostatectomy: A randomized controlled trial. J. Phys. Ther. Sci..

[9] Goode P.S., Burgio K.L., Johnson T.M., Clay O., Roth D.L., Markland A.D., Burkhardt J.H., Issa M.M., Lloyd L.K. (2011). Behavioral Therapy With or Without Biofeedback and Pelvic Floor Electrical Stimulation for Persistent Postprostatectomy Incontinence. JAMA.

[10] Filocamo M., Limarzi V., Del Popolo G., Cecconi F., Marzocco M., Tosto A., Nicita G. (2005). Effectiveness of Early Pelvic Floor Rehabilitation Treatment for Post-Prostatectomy Incontinence. Eur. Urol..

[11] Berghmans, Hendriks, Bø, Smith H., Bie D., Van Doorn V.W. (1998). Conservative treatment of stress urinary incontinence in women: A systematic review of randomized clinical trials. Br. J. Urol..

[12] Melling C.V., Goyal A. (2020). Current pharmacological management of idiopathic overactive bladder in children in the UK: A national survey of practice. J. Pediatr. Urol..

[13] Alós R., Solana A., Ruiz M.D., Moro D., García-Armengol J., Roig-Vila J.V. (2005). Novel techniques in the treatment of anal incontinece. Cir. Esp..

[14] Zhao S., Mehta A.S., Zhao M. (2020). Biomedical applications of electrical stimulation. Cell. Mol. Life Sci..

[15] Reis B.M., da Silva J.B., Rocha A.P.R., Liebano R.E., Driusso P. (2021). Intravaginal electrical stimulation associated with pelvic floor muscle training for women with stress urinary incontinence: Study protocol for a randomized controlled trial with economic evaluation. Trials.

[16] Jha S., Walters S.J., Bortolami O., Dixon S., Alshreef A. (2018). Impact of pelvic floor muscle training on sexual function of women with urinary incontinence and a comparison of electrical stimulation versus standard treatment (IPSU trial): A randomised controlled trial. Physiotherapy.

[17] De Sousa H.A., Silva M.D.G.D., Barbosa K.D.P., Vianna L.M.D.S., Pacheco Y.G., De Godoy J.R.P., Kuckelhaus S.A.S. (2017). Electrical stimulation structurally affects the tissues of the rectum and anus of nulliparous rats. J. Anat..

[18] Hernandez-Reynoso A.G., Corona-Quintanilla D.L., López-García K., Horbovetz A.A., Castelán F., Zimmern P., Martínez-Gómez M., Romero-Ortega M.I. (2021). Targeted neuromodulation of pelvic floor nerves in aging and multiparous rabbits improves continence. Sci. Rep..

[19] Mallmann S., Ferla L., Rodrigues M.P., Paiva L.L., Sanches P.R., Ferreira C.F., Ramos J.G.L. (2020). Comparison of parasacral transcutaneous electrical stimulation and transcutaneous posterior tibial nerve stimulation in women with overactive bladder syndrome: A randomized clinical trial. Eur. J. Obstet. Gynecol. Reprod. Biol..

[20] Pagani R.N., Kovaleski J.L., de Resende L.M.M. (2018). Avanços na composição da Methodi Ordinatio para revisão sistemática de literatura. Ciência Da Inf..

[21] Yang S., Sang W., Feng J., Zhao H., Li X., Li P., Fan H., Tang Z., Gao L. (2017). The effect of rehabilitation exercises combined with direct vagina low voltage low frequency electric stimulation on pelvic nerve electrophysiology and tissue function in primiparous women: A randomised controlled trial. J. Clin. Nurs..

[22] Sönmez R., Yıldız N., Alkan H. (2021). Efficacy of percutaneous and transcutaneous tibial nerve stimulation in women with idiopathic overactive bladder: A prospective randomised controlled trial. Ann. Phys. Rehabil. Med..

[23] Jacomo R.H., Alves A.T., Lucio A., Garcia P.A., Lorena D.C.R., de Sousa J.B. (2020). Transcutaneous tibial nerve stimulation versus parasacral stimulation in the treatment of overactive bladder in elderly people: A triple-blinded randomized controlled trial. Clinics.

[24] Antônio F.I., Bø K., Pena C.C., Bueno S.M., Mateus-Vasconcelos E.C.L., Fernandes A.C.N.L., Ferreira C.H.J. (2022). Intravaginal electrical stimulation increases voluntarily pelvic floor muscle contractions in women who are unable to voluntarily contract their pelvic floor muscles: A randomised trial. J. Physiother..

[25] Feng X., Lv J., Li M., Lv T., Wang S. (2022). Short-term Efficacy and Mechanism of Electrical Pudendal Nerve Stimulation Versus Pelvic Floor Muscle Training Plus Transanal Electrical Stimulation in Treating Post-radical Prostatectomy Urinary Incontinence. Urology.

[26] Del Río-Gonzalez S., Aragon I.M., Castillo E., Milla-España F., Galacho A., Machuca J., Lara M.F., Herrera-Imbroda B. (2017). Percutaneous Tibial Nerve Stimulation Therapy for Overactive Bladder Syndrome: Clinical Effectiveness, Urodynamic, and Durability Evaluation. Urology.

[27] Zhong F., Miao W., Yu Z., Hong L., Deng N. (2021). Clinical effect of electrical stimulation biofeedback therapy combined with pelvic floor functional exercise on postpartum pelvic organ prolapse. Am. J. Transl. Res..

[28] Hwang U.-J., Lee M.-S., Jung S.-H., Ahn S.-H., Kwon O.-Y. (2020). Which pelvic floor muscle functions are associated with improved subjective and objective symptoms after 8 weeks of surface electrical stimulation in women with stress urinary incontinence?. Eur. J. Obstet. Gynecol. Reprod. Biol..

[29] Li W., Hu Q., Zhang Z., Shen F., Xie Z. (2020). Effect of different electrical stimulation protocols for pelvic floor rehabilitation of postpartum women with extremely weak muscle strength: Randomized control trial. Medicine.

[30] Elena S., Dragana Z., Ramina S., Evgeniia A., Orazov M. (2020). Electromyographic Evaluation of the Pelvic Muscles Activity After High-Intensity Focused Electromagnetic Procedure and Electrical Stimulation in Women With Pelvic Floor Dysfunction. Sex. Med..

[31] Oliveira M.C., Oliveira M., Silva H., Gomes A., Nascimento G., Marini G., Micussi M.T. (2021). Evaluation of satisfaction of pelvic floor muscle training isolated and associated with tibial nerve stimulation in women with mixed urinary incontinence: A randomized, single-blinded clinical trial. Eur. J. Obstet. Gynecol. Reprod. Biol..

[32] Mateus-Vasconcelos E.C.L., Brito L.G.O., Driusso P., Silva T.D., Antônio F.I., Ferreira C.H. (2018). Effects of three interventions in facilitating voluntary pelvic floor muscle contraction in women: A randomized controlled trial. Braz. J. Phys. Ther..

[33] Brose S.W., Bourbeau D.J., Gustafson K.J. (2018). Genital nerve stimulation is tolerable and effective for bladder inhibition in sensate individuals with incomplete SCI. J. Spinal Cord Med..

[34] Li T., Feng X., Lv J., Cai T., Wang S. (2018). Short-term Clinical Efficacy of Electric Pudendal Nerve Stimulation on Neurogenic Lower Urinary Tract Disease: A Pilot Research. Urology.

[35] Ness T.J., DeWitte C., McNaught J., Clodfelder-Miller B., Su X. (2018). Spinal mechanisms of pudendal nerve stimulation-induced inhibition of bladder hypersensitivity in rats. Neurosci. Lett..

[36] Hwang U.-J., Lee M.-S., Jung S.-H., Ahn S.-H., Kwon O.-Y. (2019). Pelvic Floor Muscle Parameters Affect Sexual Function After 8 Weeks of Transcutaneous Electrical Stimulation in Women with Stress Urinary Incontinence. Sex. Med..

[37] Min J., Li B., Liu C., Hong S., Tang J., Hu M., Liu Y., Li S., Hong L. (2017). Therapeutic Effect and Mechanism of Electrical Stimulation in Female Stress Urinary Incontinence. Urology.

